# The Guide-Board

**Published:** 1870-04

**Authors:** 


### THE GUIDE-BOARD.

A PRIMITIVE and very homely name for a handsomely bound four dollar volume of seven hundred and fifty-two pages, but the welcomest sight in all nature, when cold and weary and tired and sad, near dark, the wayworn traveller in a strange and sparsely settled country, knowing not which way to turn, is the rude sign-board, with rude letters, fastened awry to a dead tree or dilapidated, leaning post, pointing out the way which he is to travel, with the welcome announcement, " One mile to ——," his home for the night!

The " Guide-board " above alluded to is the name of a book just published. The first time we saw it, the thought came across the mind, if all that reading came from a fellow's head, there certainly could not be anything left inside; and it afforded a satisfactory explanation of the manner in which the apple got into the dumpling, and the man out of the barrel after he had made it, through the bung-hole, and is a very satisfactory showing of the truth of that popular and widely quoted rhyme,—

Little head, little wit;
Big head, not a bit."

As we gazed at the huge heap of leaves, the inquiry and calculation came across the mind, " Could I be hired at any price to read that book through, 'for notice' in the columns of the *Journal?*" and the response was short, sharp, decisive; Echo answering " Never." But we read the first article, and were so much pleased with it, and the last article, which seemed so rational that we have concluded to give them both to our readers verbatim,—" The Bible," and " Growing Old Happily;" suggesting to the reader, meanwhile, that a book which begins and ends so well must have plenty more of the same sort between the two. It certainly is safe to say that every household in the United States, able to spare four dollars, will find it a most productive investment for the present and the future age ; because its teachings will be as true and rational and practical twenty years to come, as at this moment. It is too large and too handsome a book to be sent by mail; it should be ordered through some friend, or by express. It is sold only by subscription sent to D. E. Fisk & Co., Springfield, Mass. ; to the office of *Hall's Journal of Health*, 176 Broadway, New York, Room 25, or Crittenden & McKinney, 1308 Chestnut St., Philadelphia, Pa. The full title to the book is "The, Guide-board to Health, Peace, and Competence, or the Road to a Happy Old Age." A table of contents will be sent to those who desire it

